# Influence of Functional Impairment on Prognosis in Hospitalized Elderly Patients on Dialysis

**DOI:** 10.31662/jmaj.2023-0104

**Published:** 2023-09-29

**Authors:** Kouichi Tamura, Yu Soma, Tatsuya Haze, Yusuke Kobayashi

**Affiliations:** 1 Department of Medical Science and Cardiorenal Medicine, Yokohama City University Graduate School of Medicine, Yokohama, Japan; 2 YCU Center for Novel and Exploratory Clinical Trials (Y-NEXT), Yokohama City University Hospital, Yokohama, Japan

**Keywords:** Aging, Chronic kidney disease, Dialysis, Functional disability, Real-world data

Non-communicable diseases (NCDs) such as hypertension, diabetes, dyslipidemia, and visceral obesity increase the risk of developing cardiovascular diseases including myocardial infarction and heart failure, and chronic kidney disease. In addition, due to the progress of aging in recent years, the number of elderly patients with chronic kidney disease as a major complication of these NCDs is increasing compared to CKD patients without these complications of NCDs.

Recent large-scale real-world data analyzes in Japan have shown that the presence of CKD or HF in addition to diabetes exacerbates the risk of death ^(1), (2)^. In addition, in a large-scale cohort study of CKD patients in Japan, it has been reported that heart failure is more frequent than myocardial infarction and cerebrovascular disease as complications of CKD patients ^(3), (4)^. CKD complications with risk of acute kidney injury and cardiovascular-kidney comorbidity (i.e., cardiorenal syndrome with heart failure and CKD) are recent pathological features ^(5)^.

In addition, the aging of chronic dialysis patients has become also noticeable in recent years, and the recent statistical analysis survey results by the Japanese Society for Dialysis Medicine [the annual report of The Japanese Society for Dialysis Therapy Renal Data Registry (JRDR)] also showed an increasing trend in chronic dialysis patients aged 70 and over ^(6)^.

Particularly, patients undergoing chronic dialysis are reported to have the high mortality and hospitalization rates. Functional dependence is clinically recognized as a contributing factor to subsequent disability, recurrent hospitalization, and increased mortality. An increasing burden of functional dependence has been observed in several studies with progressive deterioration of kidney function, and functional dependence may contribute to both morbidity and mortality inpatients on chronic dialysis, suggesting a problem from the socio-medical perspective point of view in Japan and other countries ^(7)^.

In this issue of JMA Journal, Mandai S et al investigated the effect of the number of ADL disabilities on in-hospital outcome for elderly admitted patients aged 65 years and over on chronic dialysis using the Japanese nationwide observational cohort data ^(8)^. The authors showed that admission functional decline substantially increases in-hospital mortality, length of stay, and costs ^(8)^. From these findings, the authors concluded that routine assessment and care of functional status are essential to improve the in-hospital outcome of dialysis patients ^(8)^. The present study was well performed and the results were important and interesting especially for the practitioners and researchers in the fields of kidney medicine, gerontology, and dialysis therapy.

Nevertheless, since the present study is the observational study, the study only clarified the possible association, but not causality, between ADL disability and in-hospital outcomes in elderly dialysis population. Apparently, further interventional studies are warranted to investigate whether therapeutic interventions to improve ADLs would result in improved prognostic outcomes.

## Article Information

### Conflicts of Interest

None

### Disclaimer

Kouichi Tamura is one of the Editors of JMA Journal and on the journal’s Editorial Staff. He was not involved in the editorial evaluation or decision to accept this article for publication at all.
